# Medical and Philosophical News

**Published:** 1781-04

**Authors:** 


### SECTION III.

### Medical and Philosophical News.

THE Royal Medical Society at Paris have propofed the following queftion for a prize of fix hundred livres: "What are the means "of preventing the evils to which children are "expofed during dentition, or of remedying "them when they have actually taken place?" The differtations on this fubject are to be written either in French or Latin, and fent to M. Vicq. D'Azyr, fecretary to the fociety at Paris, on or before the firft of November 1781.

The

---

The Dutch Society of Sciences at Harlem, have propofed the following fubject for a gold medal: 1ft, "What are the different fpecies of "fluids that appear to be air, and to which "the names of fixed air, dephlogifticated air, "inflammable air, nitrous air, acid air, alkaline "air, &c. are given; and in what refpects do "they differ from each other, and from atmo-"fpheric air? 2dly, Have each of thefe kinds "of elaftic fluid fufficient affinity with at-"mofpheric air to deferve to be confidered "as a fpecies of air? 3dly, How far can we "determine the nature of atmofpheric air by "experiments and obfervations made with "fluids?" The differtations on this fubject are to be fent to the fecretary of the fociety at Harlem, before the firft of January 1782.

---

Dr. Brownrigg, F. R. S. of Ormithwaite in Cumberland (who is well known to the lovers of philofophical chemiftry by feveral ingenious publications) in a letter to one of his friends, a celebrated phyfician in London, fays, that he feveral

ſeveral years ago diſcovered a method of converting a moſt light, ſubtile, elaſtic, inviſible fluid (namely, air) into a ponderous, palpable, viſible body; in which form, without any addition, he can exhibit it to the eye, and this in great abundance. This promiſes to be a capital diſcovery, leading to others of great moment; "for which reaſon"—adds the doctor in his letter—"I have ſat brooding over it theſe "five-and-thirty years." Being unwilling that this diſcovery ſhould die with him, he intends to publiſh his obſervations and experiments on this ſubject, or at leaſt a conſiderable part of them, next winter.

---

The late Dr. Fothergill, a little before his death, was preſent on a ſurvey at Buxton with two other phyſicians. As the Duke of Devonſhire means to make ſome improvements at that place, they were deſirous of propoſing to his Grace, ſuch as might prove conducive to health. — At preſent there are only two baths at Buxton, one for gentlemen, the other for ladies; and four perſons only can bathe in each at a time. The rule for bathing is to ſet apart the firſt hour for the gentlemen and ladies who lodge lodge in the houſe, the ſecond for thoſe who lodge in the town, and the third for the inhabitants. The preference thus given to thoſe who lodge in the houſe, very often occaſions ſo many to be crowded together in one apartment, that they ſuffer as much from this circumſtance, as they gain benefit from bathing. The ſcheme in agitation, therefore, is for every houſe to be furniſhed with a bath, that it may be no longer a monopoly.—As the natural heat of the Buxton water is only about 80° of Fahrenheit's thermometer, Dr. Fothergill propoſed, as a farther improvement, that it ſhould be heated artificially to about 90°, in order to render it more efficacious in cutaneous diſeaſes.

---

At a public meeting of the Royal Medical Society of Paris on the 6th of March, at their apartments in the Louvre, M. Lorry read a diſcourſe on the odours of medicines, divided into five claſſes; M. Carrere communicated the plan of a new work on the mineral waters of France; M. Defourcroy delivered ſome obſervations on a new method of employing certain reagents in the analyſis of mineral waters; M.

Vicq.

Vicq. d'Azyr, ſecretary to the ſociety, pronounced the eulogium of the late M. Navier, phyſician at Chalons ſur Marne, and fellow of the ſociety; M. Caille gave an account of ſome chymical inquiries on the different proceſſes employed for preparing emetic tartar; the Abbé Teſſier read a paper on a diſeaſe which he calls "la maladie rouge," and which has lately proved fatal to a prodigious number of ſheep in the diſtrict of Sologne; the buſineſs of the meeting ended with the reading of a paper by M. Mauduyt on the effects of electricity, applied to incubation and vegetation.

---

Mr. John Elliott, the ingenious author of philoſophical obſervations on the ſenſes of viſion, &c. is employed in collecting and publiſhing the medical works of the late Dr. Fothergill, in one volume 8vo. with an account of his life, and occaſional notes.

---

Mr. Thomas Henry of Mancheſter, F. R. S. is about to publiſh a work, entitled, "An Account of a cheap and eaſy method of preſerving Water at ſea from Putrefaction, with-

" out injury to its original pleaſantneſs and pu-

" rity : to which is added, a mode of impreg-

" nating water in large quantities with fixed

" air for medicinal uſes, on ſhipboard and in

" hoſpitals ; and likewiſe a proceſs for the pre-

" paration of artificial yeaſt."

---

To the account already given of the late M. Lieutaud, in our Journal for February (page 138) we are now enabled to add the following particulars of the life of that celebrated phyſician — Joſeph Lieutaud was born at Aix in 1703. He was the youngeſt of twelve children, and was naturally of a weakly conſtitution. He was educated under his uncle Gabriel Lieutaud, who acquired ſome reputation as a botaniſt. After taking a doctor's degree at Aix, he ſpent ſeveral years at Montpelier, and from thence returned to his native city, where he procured the appointments of Profeſſor of Phyſic, and Phyſician to an hoſpital, and ſoon came into extenſive practice. His houſe became the rendezvous of men of letters, who met there on ſtated days to converſe on literary ſubjects. The famous Marquis d'Argens uſed ufed often to affift at thofe meetings, and ever after profeffed a friendfhip for M. Lieutaud.

About the year 1750, he was called from Aix to Verfailles to act as phyfician to the Royal Infirmary, and this circumftance may be confidered as the foundation of his future good fortune. While he officiated in that capacity, he diffected a great number of bodies, and publifhed his obfervations on the heart and bladder. He was afterwards appointed phyfician to the princes of France, and in 1774 firft phyfician to the King, a poft not only of great honour, but of confiderable emolument, the profits of it being eftimated at upwards of £ 3000 fterling per annum. His uncle Gabriel had bequeathed him his library which was confiderable. M. Lieutaud, by the additions he made to it rendered it very valuable, and Monfieur, the King's eldeft brother, gave him a confiderable fum for it, and left him the free poffeffion of it during life. M. Lieutaud lived in a frugal and temperate manner, and died fuddenly.

Befides the works we formerly mentioned, he was author of the following; viz. *Elementa phyfiologiæ, juxta folertiora, noviffimaque phyficorum experimenta et accuratiores anatomicorum obfervationes,*

*ſervationes, concinnata.* Amſtelodam. 8vo. 1749. *Precis de la matiere medicale*, 8vo. Paris, 1766. He was likewiſe the author of ſeveral anatomical papers, printed in the Memoirs of the Royal Academy of Sciences for 1752, 1753, and 1754.

